# Long Telomeres Do Not Affect Cellular Fitness in Yeast

**DOI:** 10.1128/mBio.01314-17

**Published:** 2017-08-29

**Authors:** Yaniv Harari, Shira Zadok-Laviel, Martin Kupiec

**Affiliations:** Department of Molecular Microbiology and Biotechnology, Tel Aviv University, Ramat Aviv, Israel; Harvard Medical School

**Keywords:** aging, cancer, ethanol, fitness, telomere, yeasts

## Abstract

Telomeres, the ends of the eukaryotic chromosomes, help to maintain the genome’s integrity and thus play important roles in aging and cancer. Telomere length is strictly controlled in all organisms. In humans, telomeres shorten with age, and it has been proposed that telomere shortening may play a causal role in aging. We took advantage of the availability of yeast strains with genetically or physiologically generated differences in telomere length to measure the effect that telomere length may have on cellular growth. By comparing the growth rates affecting telomere length of various yeast mutants we show that there is no correlation between their telomere length and cellular fitness. We also show that wild-type yeast cells carrying extremely long telomeres (~5 times longer than the average) showed no signs of mitotic or meiotic defects, and competition experiments found no differences in growth between strains with normal telomeres and strains with long telomeres. No advantage or disadvantage of cells with long telomeres was detected under stress conditions either. Finally, telomere length had no effect in a chronological life span assay, which measures survival of post-mitotic-stage cells. We conclude that extreme telomere length has no effects (positive or negative) on the fitness of yeast cells.

## IMPORTANCE

Telomeres protect the chromosomal ends from fusion, degradation, and unwanted repair. Therefore, telomeres preserve genome stability and cell viability. In humans, telomeres shorten with each cell duplication event and with age. It has thus been proposed that telomere shortening may be responsible for human aging and that elongation of telomeres may be a way to rejuvenate cells and to combat aging. However, it is difficult to prove this hypothesis in human cells. Yeasts are easy to manipulate and have telomeres whose length is strictly maintained. Here we show that yeast cells manipulated to have extremely long telomeres (~5-fold those of normal cells) did not show any improvement or reduction in fitness compared to otherwise identical cells with telomeres of normal length under all the conditions tested. Moreover, an assay that measures cell aging showed no effect of the presence of extremely long telomeres. We thus conclude that extreme telomere length, at least in yeast cells, does not affect cellular fitness, aging, or senescence.

## INTRODUCTION

Telomeres are nucleoprotein structures located at the ends of chromosomes and are essential for chromosome function, replication, and stability. Telomeres protect chromosome ends and prevent them from being misidentified as double-strand breaks, which could lead to undesired chromosomal rearrangements (1, 2). Telomere length regulation is central to telomere function. In most eukaryotes, the telomeric DNA consists of tandem repeat tracts whose overall lengths differ between organisms but are always highly regulated (3). This telomeric DNA sequence is synthesized by a ribonucleoprotein enzyme named telomerase, a reverse transcriptase that carries its own RNA template (4). Much of what we know about the maintenance of telomere length comes from studies in simple organisms, such as yeast (5, 6). Genome-wide studies in yeast have uncovered a large number of genes (~500) that affect telomere length maintenance. These *TLM* genes encode proteins with various biochemical activities and cellular locations which affect a number of cellular processes (7–10).

Cells in all organisms are constantly exposed to environmental changes and stressful perturbations that affect them in many ways. The cellular response to environmental challenges has been intensively studied in several model organisms (11–14). Despite the advances in our understanding of the mechanisms that respond to external inputs, the long- and short-term effects of various stresses are largely unknown. Previously, we studied the effect of different environmental stresses on telomere length in the budding yeast *Saccharomyces cerevisiae* (15). The majority of environmental stresses do not alter telomere length. However, exposure to several specific environmental stress factors such as ethanol or acetic acid significantly induces telomere elongation, whereas others (e.g., caffeine) result in telomere shortening (16).

Telomere length regulation is central to telomere and cell function (17). Telomere length is associated with cell senescence and longevity (18, 19), as well as with age-related disorders and cancer (20). In higher eukaryotes, telomerase is highly expressed, mainly at the early stages of development (in embryonic stem cells) (21, 22). In somatic cells, however, telomerase expression is low and telomeres shorten with each cell division (23, 24). This progressive telomere shortening represents a “molecular clock” that underlies cellular aging (20, 25). Reactivation of telomerase in cultured cells results in an extended life span, leading to their apparent immortalization (26). When yeast cells that lack telomerase activity are cultured, there is a progressive decline in the fraction of growing cells to less than 1%, followed by the selection of a small subpopulation of cells that maintain telomeres via a recombination-mediated pathway (27). Each organism maintains tight homeostasis regarding telomere length, suggesting that deviation from a set telomere length has a detrimental effect on fitness. Whenever telomeres shorten or telomerase activity is reduced or absent, cells do indeed show reduced fitness and are in danger of senescence. However, not a lot is known about the potential fitness cost of maintaining longer-than-wild-type (wt) telomeres. The existence of mechanisms that shorten extremely elongated telomeres such as TRD (telomere rapid deletion) in yeast (28) and telomere trimming in mammalian cells (29) suggests that long telomeres may be disadvantageous for cell fitness. In this study, we systematically compared wt and mutant yeast strains carrying long telomeres to isogenic strains of normal telomere length. Our results show no indication of a fitness cost even for extremely long telomeres.

## RESULTS

**FIG 1:**
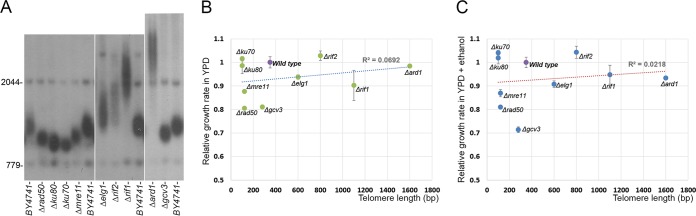
The effect of *tlm* mutations on cell fitness. (A) Representative Telo-blot analysis of the different *tlm* mutants. (B) Relative growth rate measurements for different *tlm* mutants in YPD medium. For each strain, the growth rate was normalized to that of the wild-type (BY4741) strain (whose rate is shown with a value of 1). The different *tlm* strains are shown on the *x* axis according to their telomere length phenotypes. (C) A similar graph for strains grown in YPD–5% ethanol medium.

A very tightly maintained homeostatic mechanism keeps telomeres of all organisms in a fixed size range. It is not clear whether having elongated telomeres is advantageous or disadvantageous for a cell. In theory, if an optimal length exists for each organism, cells harboring long telomeres could have difficulties in properly maintaining their telomeres for a long period of time and long-telomere cells could thus experience telomere dysfunction and/or chromosomal end-to-end fusions (30, 31). On the other hand, it has been suggested that elongating telomeres could rejuvenate cells, setting their biological clock back and thus improving their fitness. By systematically screening yeast mutant collections, we have identified a large number of yeast mutants (~500) affected in telomere length maintenance (*tlm* mutants). About half of them show shorter-than-wt-length telomeres, and the other half show long telomeres (7, 10, 32). One way to examine the effect of elongated telomeres on cell viability is by following the growth rate of different *tlm* mutants. The problem with this method is that it is impossible to clearly distinguish between the fitness effect caused by the mutation (which may affect several processes in the cell in addition to telomeres) and the fitness effect caused by the elongated telomeres. However, if we combine growth rate and telomere length data of a large number of *tlm* mutants, we can still ask whether there is a correlation between telomere length and cell fitness. We measured the growth rates of different long and short *tlm* mutants in yeast extract-peptone-dextrose (YPD) media at 30°C and the lengths of their telomeres (Fig. 1A and B). As can be seen in Fig. 1, although some *tlm* mutations had no effect on growth rate and others did extend cell division time (compared to the wt control), there was no correlation between telomere length and cell division time, taken here as a proxy for cellular fitness (*R*^2^ = 0.0692).

Different external stress conditions can affect telomere homeostasis and thereby change telomere length. In yeast, exposure to low concentrations of ethanol leads to extensive telomere elongation mediated by a physiological mechanism (15). We followed the growth rate of different *tlm* mutants, this time under conditions of growth in YPD media with 5% ethanol. Again, a distribution of telomere sizes and cell division times can be observed (Fig. 1C), but no correlation is seen (*R*^2^ = 0.0218). We conclude that among *tlm* mutants grown under favorable conditions or under stress conditions, telomere length does not seem to have a strong effect on fitness.

**FIG 2:**
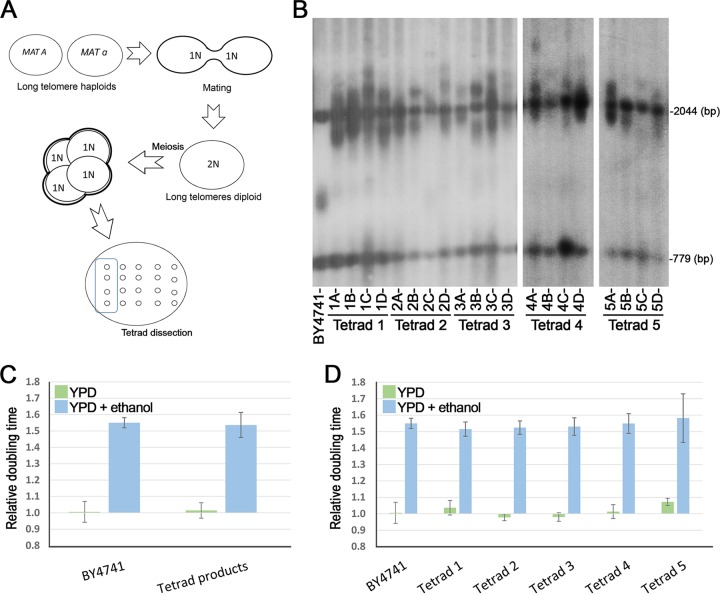
Cell fitness in wild-type strains with long telomeres. (A) Schematic cartoon representing the process of mating of haploid cells, followed by sporulation and tetrad dissection. (B) Telo-blot analysis of haploid meiotic products with long telomeres. (C) Relative doubling time measurements for untreated wild-type cells (BY4741) and haploid meiotic products of long telomeres in media consisting of either YPD (green bars) and YPD plus 5% ethanol (blue bars). For each candidate, doubling times in YPD media and in YPD–5% ethanol media are normalized according to the doubling time of BY4741 in YPD medium. (D) Relative doubling-time measurements for wild-type cells (BY4741) of normal telomere length and haploid meiotic products from a single tetrad in media consisting of either YPD (green bars) or YPD plus 5% ethanol (blue bars) (results were normalized as described for panel C).

Since ethanol exposure leads to extensive telomere elongation by a physiological mechanism, we were able to create genetically identical yeast wild-type strains of different telomere lengths. Haploid yeast strains of mating type a (BY4741) or alpha (BY4742) were grown in rich medium containing 5% ethanol for 400 generations. At this stage, the strains exhibited extremely elongated telomeres (~1,900 nucleotides [nt] in length rather than ~350; see Fig. S1A in the supplemental material). Since long-term exposure to ethanol could also lead to the accumulation and fixation of rare genetic events such as aneuploidy and mutations within the population (33–35), we mated the long-telomere haploid strains and subjected them to meiosis. Tetrad analysis separated individual haploid spores, and telomere Southern blot (Telo-blot) analysis confirmed that all carried long telomeres (Fig. 2A and B). The extremely long telomeres of these strains did not impair the meiotic divisions, and the viability of the spores was 97%, comparable to that of a control isogenic diploid of normal telomere length (96%). When we calculated the average growth rate of all the new haploid cells (Fig. 2C) or of all four haploid products from each meiosis (Fig. 2D [Tetrad 1 to Tetrad 5]) in medium containing 5% ethanol, we were unable to detect any significant growth rate advantage or disadvantage of these cells with long telomeres compared to the isogenic wild-type strain. This was true under optimal growth conditions and also for the results seen with medium containing ethanol (Fig. 2C and D). These results imply that long telomeres do not improve cell fitness under either optimal growth conditions or ethanol stress conditions. On the other hand, the results show that maintaining extremely long telomeres does not inflict any fitness disadvantage either, implying that the presence of long telomeres does not lead to telomere dysfunction, replication stalling, or cell cycle arrest. Flow cytometry (FC) results from cultures of cells with normal or long telomeres support this observation (Fig. S1B).

10.1128/mBio.01314-17.1 FIG S1  (A) Telo-blot analysis of wild-type cells with either normal or very long telomeres. BY4741 is shown in the first lane and is compared to BY4741 (*MATA*) and BY4742 (*MAT*α) wild-type cells that were exposed for 400 generations to 0.05 ethanol. (B) Flow cytometry cell cycle progression analysis of logarithmic-growth-phase wild-type cells with either normal telomeres (orange) or very long telomeres (blue for BY4741, purple for BY4742). The two peaks correspond to cells in the G_1_ and G_2_ cell cycle stages. Download FIG S1, PDF file, 1.5 MB. Copyright © 2017 Harari et al. 2017 Harari et al. This content is distributed under the terms of the Creative Commons Attribution 4.0 International license.

**FIG 3:**
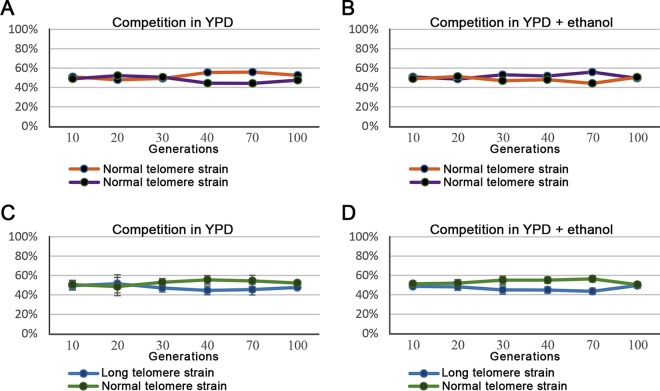
Competition experiments. (A) Assay of competition between two differently marked wild-type cells of normal telomere length grown in YPD medium for 100 generations. Cell type abundance is presented in intervals of 10 generations. (B) Assay similar to that described for panel A, performed using YPD plus 5% ethanol. (C) Competition assay between two isogenic wt strains, one of normal telomere length, and one with long telomeres. (D) Assay similar to that described for panel C, performed using YPD plus 5% ethanol.

To rule out the possibility that a small effect on fitness was present but not detected in our growth rate experiments, we also performed direct competition assays. We inserted either the *KanMX* cassette or the *HygMX* cassette at the *HO* gene (*ho*::*KanMX* or *ho*::*HygMX* strains; deletion of this locus has no effect on growth) of cells with either normal or long telomeres. Then, cells with different endogenous markers were mixed in the same tube in equal amounts and were grown together for 100 generations. If one of the strains had even a slight growth advantage (perhaps one that would be undetectable in monitoring its individual growth), its abundance within the population would be expected to increase with time. As a control, the same competition assay was performed with two unstressed wild-type strains carrying either the *ho*::*KanMX* marker or the *ho*::*HygMX* marker to rule out any effect of the cassettes themselves (Fig. 3A and B). After 100 generations in media consisting of either YPD or YPD plus 5% ethanol, we could see no effect of telomere length on fitness (Fig. 3C and D). These results support our previous observations and confirm that long telomeres do not cause any fitness advantage or disadvantage.

**FIG 4:**
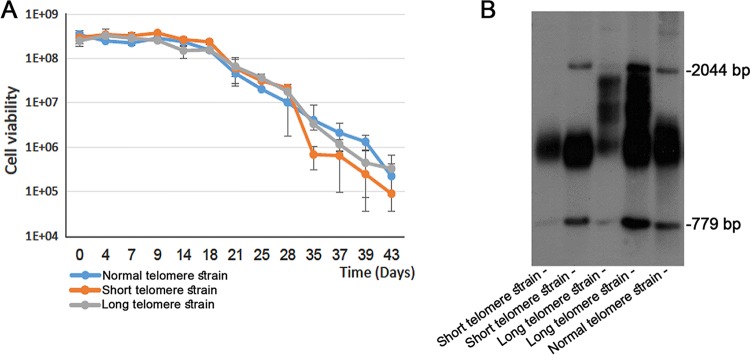
The effect of telomere length on chronological life span (CLS). (A) Chronological life span (CLS) assay of isogenic wild-type strains of yeast cells of normal telomere length (blue), short telomere length (orange), and long telomere length (gray). For each strain, cell viability (measured as the number of CFU per milliliter) is presented as a function of time. (B) Telo-blot analysis of the surviving cells within the senescent CLS cultures after day 43.

As previously discussed, short telomere length in humans is associated with cellular senescence, longevity, aging, age-related disorders, and cancer (18–20, 23, 36, 37). In order to determine if telomere length indeed affects cellular senescence in yeasts as well, we measured the chronological life span (CLS) of wild-type cells with normal, long, or short telomeres. CSL data represent the survival rates of cells after they have stopped dividing (thus mimicking neuronal human cells; see, for example, reference 38). In this assay, cells are allowed to grow and reach stationary phase and are then kept under the same conditions for a long period of time. At various time intervals, the viability of the cultures is monitored by plating the cells on rich medium. This method allows us to calculate the mean and maximum survival times of nondividing yeast populations. Strikingly, no significant change in cellular senescence rates was observed between cells with normal telomeres and cells with long telomeres (Fig. 4A), implying that the presence of long telomeres does not affect chronological senescence. For the strain with short telomeres (obtained by growing the wt strain in the presence of caffeine for 100 generations [15]), a modest reduction in cellular viability was observed after day 28 in comparison to the results seen with the strains with normal and long telomeres. At this stage, however, more than 95% of the cells within the population had already lost the ability to undergo cell division. Interestingly, even after 43 days of incubation, when less than 0.1% of the cell population survived, the surviving long-telomere cells still kept their long-telomere phenotype (Fig. 4B).

**FIG 5:**
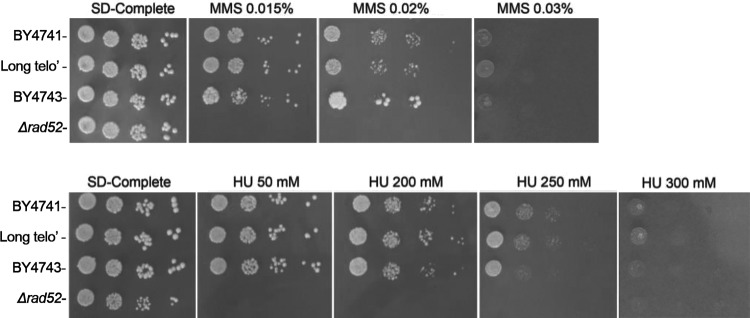
Spot assay (10-fold dilutions) of isogenic wild-type strains with normal and long telomeres on plates containing methyl methanesulfonate (MMS) or hydroxyurea (HU). The diploid BY4743 strain and the Δ*rad52* strain are shown as controls.

Next, we asked if telomere length can affect yeast sensitivity to DNA damage and chronic replicative stress. Cells with normal or long telomeres were serially diluted and plated on plates containing various concentrations of methyl methanesulfonate (MMS), a DNA-damaging agent, or of hydroxyurea (HU), a replication-stressing agent (Fig. 5). Colonies were counted after 3 days of incubation. In both MMS and HU, we saw no differences in sensitivity between the cells with long telomeres and the cells with normal telomeres (Fig. 5). We thus conclude that telomere length has no effect under conditions of replicative or genotoxic stress either.

## DISCUSSION

Telomere integrity is required for cell proliferation and cell viability. Telomere shortening is considered one of the best-characterized mechanisms of cellular aging (39), since it correlates with the onset of replicative senescence (36). Telomeres also shorten in humans with age, and abnormally short telomeres recapitulate several premature aging phenotypes (40). It has been proposed that the decrease in telomere length seen with the successive rounds of DNA replication that accompany mitotic division plays a causal role in the aging process. However, distinguishing between a correlative relationship of telomere length with aging and an alternative view (i.e., that telomere shortening is the cause of aging) is extremely difficult, particularly in humans. Human syndromes associated with short telomeres vary in severity and age onset. The most severe affect children and may result in early death. Diseases associated with short telomeres present a characteristic spectrum in which fast-dividing tissues are affected the most. Bone marrow failure and abnormalities in the skin, mucosa, and nails are common first manifestations, but some patients have intestinal villous atrophy, immunodeficiency, developmental deficiencies, and pulmonary fibrosis (40). The life spans of these patients are usually short, and it seems clear that short telomeres are associated with reduced fitness. Although human cases with long telomeres have been identified, in all instances these were cancer patients, and thus it is not possible to draw conclusions about a possible longevity-promoting effect of having long telomeres in humans (summarized in reference 40).

Extrapolation of yeast results to humans is complicated by the fact that patients with short-telomere syndromes exhibit manifestations that (as in the case of *tlm* yeast mutants) may be due only partially to the effect of the mutations on telomere length. Further studies, with larger populations, will be necessary to clarify this point. On the other hand, it has been known for a very long time that telomere length is highly heterogeneous within every age class in the normal population and that this is the case in spite of the existence of a very robust length homeostasis mechanism at the individual level. Therefore, some healthy individuals of a given age carry long (sometimes quite long) telomeres and others carry short (sometimes quite short) telomeres. One can imagine that the amplitude of the variation in telomere length in every class would tend to decrease with age if carrying physiologically short or long telomeres decreased fitness. In fact, this is observed only in members of the class of very old (>90 years) individuals (41). Intriguingly, that does look very much like the very late effect of short telomeres on the yeast chronological life span seen in this study (Fig. 4A). On the other hand, correlative studies found longer telomeres and better health among centenarians, a fact that led to the assumption that long telomeres may provide a fitness advantage (42).

As a unicellular eukaryote, *S. cerevisiae* has the advantage of being the simplest and shortest-lived organism among the major eukaryotic aging model systems and thereby provides a useful system to examine the effects of telomere length dysregulation on cell viability.

In this study, we showed that yeast cells are extremely tolerant of telomere length variation. This result is not trivial since telomere length in yeasts is under strict size control, with a large number of genes involved in its homeostasis and a stable average size that shows relatively low variability (see Fig. S1A in the supplemental material). The fact that yeast cells can maintain telomeres that are ~5 times longer than normal without showing signs of growth defects is surprising. Our results thus show that long telomeres are able to recruit the normal telomere-bound proteins, assume their normal packaging within the nucleus, and function both during DNA replication and to protect the chromosome ends. We also found no evidence of a disadvantage associated with the presence of long telomeres during meiosis or under stressful situations. Previous studies in *Kluyveromyces lactis* (43) also failed to reveal any effect of hyperelongated telomeres in meiosis. In other words, extremely long telomeres are perfectly functional, at least under the conditions tested in this study (it is possible that under some particular conditions of stress, or if elongation were to exceed a certain threshold, a fitness defect could become apparent). Moreover, the kinetics of aging of yeast cells with long telomeres is very similar to that of cells with normal telomere length, suggesting that telomere length is not involved in determining the yeast chronological life span, which mimics aging in postmitosis human cells, such as neurons. Future experiments should explore whether telomere length has an effect on replicative life span (the number of cell divisions attainable before cellular senescence and death), which mimics aging in stem cells.

## MATERIALS AND METHODS

### Yeast strains.

All strains described in this article are derivatives of BY4741, BY4742, and BY7473 (Table 1).

**TABLE 1:** Yeast strains used in the study

Strain	Phenotype	Reference or source
BY4741	*MATA Δura3 Δmet15 Δleu2 Δhis3*	44
BY4742	*MATalpha Δura3 Δlys2 Δleu2 Δhis3 ho*::*KanMX*	44
BY4743	*Mat**a***/*MATalpha Δura3*/*Δura3 Δleu2*/*Δleu2 Δhis3*/*Δhis3 Δmet15*/+ +/*Δlys2*	44
*ByA-hoKanMX*	*MAT*a *Δura3 Δmet15 Δleu2 Δhis3 ho*::*KanMX*	This study
*ByA-hoHygMX*	*MAT*a *Δura3 Δmet15 Δleu2 Δhis3 ho*::*HygMX*	This study
*Byɑlpha-hoKanMX*	*MATalpha Δura3 Δlys2 Δleu2 Δhis3 ho*::*KanMX*	This study
*Byɑlpha-hoHygMX*	*MATalpha Δura3 Δlys2 Δleu2 Δhis3 ho*::*HygMX*	This study

### Growth media.

The YPD medium (yeast rich medium) consisted of 1% Bacto yeast extract (Difco), 2% Bacto peptone (Difco), and 2% glucose. Any other stressing agent used was added according to its final concentration in the YPD medium. For selection, 200 mg/liter G418 geneticin (CalBioChem) or 200 mg/liter hygromycin B (Invitrogen) was added. The YPD-plus-ethanol medium was normal YPD medium that also contained 5% ethanol. The SD Complete medium (yeast defined medium) consisted of 0.67% Bacto yeast nitrogen base without amino acids (Difco) and 2% glucose. All amino acids and nucleobases were added according to requirements.

### Flow cytometry.

A 200-μl volume of a logarithmic cell culture (optical density at 600 nm [OD_600_] of 0.6) was harvested and resuspended in 60 µl of 50 mM Tris (pH 7.5), and 140 µl of ethanol was added; cells were then kept overnight (ON) at 4°C. Fixed cells were centrifuged and washed once in 200 µl of 50 mM Tris (pH 7.5) buffer and resuspended in 100 µl RNase (0.2 mg/ml)–50 mM Tris (pH 7.5) for 2 h at 37°C. Proteinase K (0.2 mg/ml)–50 mM Tris (pH 7.5) was added to each tube, and cells were incubated for 60 additional minutes at 50°C. A 20-μl volume of the sample was taken into a new tube, and a 180-µl volume of 18 µg/ml propidium iodide–50 mM Tris (pH 7.5) was added. The samples were kept in the dark at 4°C ON, sonicated twice at a low setting (20% power) for 3 to 5 s, and stored in the dark at 4°C. A flow cytometry MACSQuant system was used for reading the results. Results were analyzed using either Flowing software or the FlowJo program.

### Telomere Southern blot (Telo-blot) analysis.

A 2.5-µg volume of genomic DNA was digested with XhoI and incubated for 16 h at 37°C. The DNA was separated on a 1% agarose gel and blotted onto a Nytran nylon membrane. The membranes were hybridized to an *S. cerevisiae*-specific telomeric probe and size-control fragments (15). Hybridizations were carried out overnight in Church buffer (1% bovine serum albumin [BSA], 0.5 M buffer phosphate, 1 mM EDTA, 7% SDS) (30 ml), and the reaction mixtures were washed three times (for 20 min in 40 ml each time) with the following dilutions of ×20 SSC (1× SSC is 0.15 M NaCl plus 0.015 M sodium citrate) (0.5 M NaCl, 0.05 M C_6_H_5_Na_3_O_7_): ×2 SSC plus 0.1% SDS, ×0.2 SSC plus 0.1% SDS, and ×2 SSC. Hybridizations and washes were performed at 65°C. A Fujicom film was exposed (at −70°C) for 3 days.

### Telomere length analysis.

Telomere length was measured using the Tel-Quant program (45).

### Fitness and competition experiments.

A total of ~10^6^ cells were grown for 24 h in a Tecan Horizon robot, and OD was automatically measured every 30 min. For competition experiments, cells marked with either the KanMX or HygMX markers were mixed in a 1:1 ratio and grown as described above.
