# Supplementary figures and images for: Long Telomeres Do Not Affect Cellular Fitness in Yeast
Source: mBio. 2017 Aug 29;8(4):e01314-17. doi: 10.1128/mBio.01314-17 (PMC5574717; doi:10.1128/mBio.01314-17)

**A**

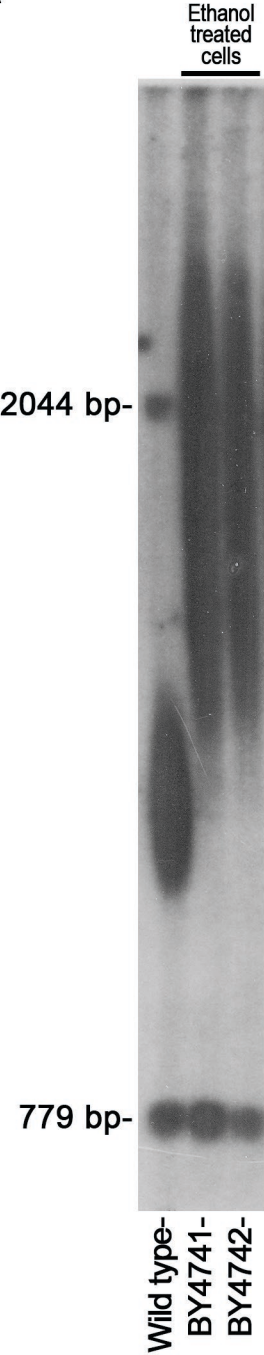

**B**

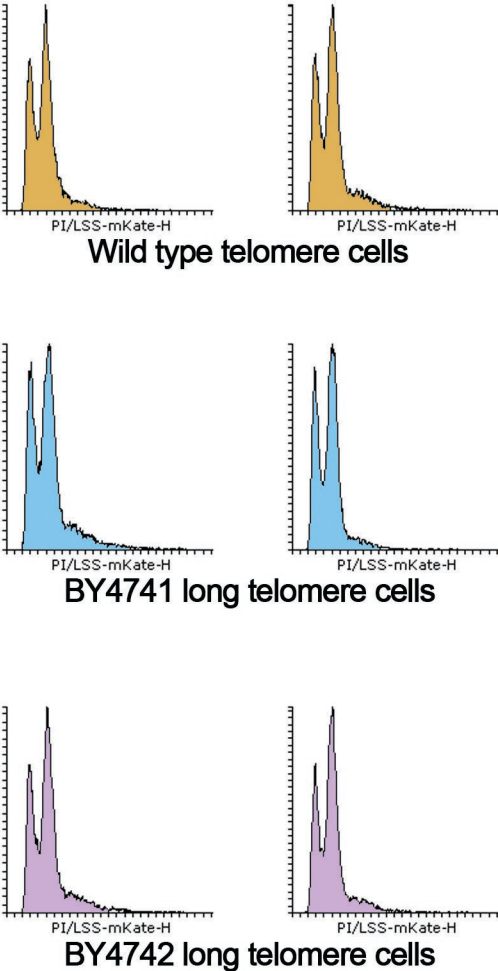

Supplementary Figure 1

Supplement: FIG S1 [file mbo004173461sf1.pdf]
